# Medical residents reflect on their prejudices toward poverty: a photovoice training project

**DOI:** 10.1186/s12909-014-0274-1

**Published:** 2014-12-31

**Authors:** Christine Loignon, Alexandrine Boudreault-Fournier, Karoline Truchon, Yanouchka Labrousse, Bruno Fortin

**Affiliations:** Family Medicine Department, Faculty of Medicine, Université de Sherbrooke (Longueuil Campus), 150 Place Charles Lemoyne, Room 200, Longueuil, QC J4K 0A8 Canada; Department of Anthropology, University of Victoria, PO Box 1700 STN CSC, Victoria, BC V8W 2Y2 Canada; Center for Oral History and Digital Storytelling (COHDS), Concordia University, 1455 de Maisonneuve Blvd W, Montreal, QC H3G 1 M8 Canada; Academic Primary Care Unit Charles-LeMoyne, 299, boulevard Sir-Wilfrid-Laurier, bureau 201, Saint-Lambert, QC J4R 2 L1 Canada

**Keywords:** Healthcare disparities, Poverty, Participatory action research, Photovoice, Residents, Education, Healthcare professionals, Medicine

## Abstract

**Background:**

Clinicians face challenges in delivering care to socioeconomically disadvantaged patients. While both the public and academic sectors recognize the importance of addressing social inequities in healthcare, there is room for improvement in the training of family physicians, who report being ill-equipped to provide care that is responsive to the living conditions of these patients. This study explored: (i) residents’ perceptions and experience in relation to providing care for socioeconomically disadvantaged patients, and (ii) how participating in a photovoice study helped them uncover and examine some of their prejudices and assumptions about poverty.

**Methods:**

We conducted a participatory photovoice study. Participants were four family medicine residents, two medical supervisors, and two researchers. Residents attended six photovoice meetings at which they discussed photos they had taken. In collaboration with the researchers, the participants defined the research questions, took photos, and participated in data analysis and results dissemination. Meetings were recorded and transcribed for analysis, which consisted of coding, peer debriefing, thematic analysis, and interpretation.

**Results:**

The medical residents uncovered and examined their own prejudices and misconceptions about poverty. They reported feeling unprepared to provide care to socioeconomically disadvantaged patients. Supported by medical supervisors and researchers, the residents underwent a three-phase reflexive process of: (1) engaging reflexively, (2) break(ing) through, and (3) taking action. The results indicated that medical residents subsequently felt encouraged to adopt a care approach that helped them overcome the social distance between themselves and their socioeconomically disadvantaged patients.

**Conclusions:**

This study highlights the importance of providing medical training on issues related to poverty and increasing awareness about social inequalities in medical education to counteract prejudices toward socioeconomically disadvantaged patients. Future studies should examine which elective courses and training could provide suitable tools to clinicians to improve their competence in delivering care to socioeconomically disadvantaged patients.

## Background

Poverty and inequities of access to primary care present serious threats to health. Socioeconomically disadvantaged persons are the least well-served in terms of healthcare services [1] and are least likely to have a family physician [2]. They report more unmet health needs than do people with higher incomes [3]. They experience negative healthcare interactions and sometimes feel judged by physicians [3-6].

Clinicians may face challenges in delivering care to socioeconomically disadvantaged persons and are not well prepared to take into account the social context to create therapeutic alliances with them. Health professionals, particularly physicians, have little understanding of these patients’ social situation. Their lack of knowledge and mistaken perceptions of poverty affect the quality of clinical interactions [7,8].

Physicians tend to be more directive with socioeconomically disadvantaged patients, spend less time with them, and provide less information concerning treatment options [9]. In a study of residents in medicine, 25% thought poverty was a consequence of laziness, 50% thought the poor were more likely to abuse the healthcare system, and 50% thought the poor were less attentive to their health than the rest of the population [10]. Primary care physicians, being close to patients’ personal and day-to-day experiences, occupy an important position with major impact on people’s lives [11].

Hence, there is a need to strengthen family medicine residents’ training and better prepare them to consider the impact of poverty on health and healthcare. Unfortunately, few medical residency programs offer satisfactory and well-resourced training programs that would prepare future family physicians to cope with poverty issues in the healthcare process. There are some innovative and promising training programs, such as Oregon Health and Science University’s (OHSU) social medicine program for homeless and addicted patients that provides seminars and experiential learning in the community [12]. While medical schools’ efforts to foster humanism and/or cultural competence are diverse and not much evaluated, three elements seem to be essential in training related to poverty: high involvement of a mentor as role model, experiential learning with supportive supervision, and time for critical reflection and discussion about the learning experience [13-15].

Photovoice is a participatory action research method that uses photography to enable participants to share experiences and develop critical consciousness of a variety of topics [16,17]. There have been very few studies using photovoice with medical residents, supervisors, and researchers in a family medicine teaching context. A brief note by Wang, Anderson, and Stern [18] explaining how they used photovoice with final-year medical students is one rare case. This elective course explored professional values and health policy issues. Students photographed aspects of healthcare delivery that could be changed through policies. Three participants presented their work to policy-makers and university representatives. Leipert and Anderson [19] used photovoice with 38 nursing students to promote and recognize the value of nurses’ care delivery in Canadian rural areas. More recently, also in nursing, Garner [20] encouraged the use of photovoice for teaching and learning, arguing specifically that photovoice facilitated students’ cultural awareness and competence in geriatric nursing. Except for such rare cases, there seems to be a gap in medical education concerning the use of photovoice as a tool to foster medical residents’ awareness of discrimination of certain patient groups, such as immigrants or socioeconomically disadvantaged persons. Photovoice may help raise medical residents’ awareness and increase their skills for delivering care to patients from these groups [21].

This participatory study conducted in a primary care teaching unit in Quebec, Canada, explored residents’ perceptions and experience in relation to providing care for socioeconomically disadvantaged persons and examined how their participation in a photovoice study, including in a one-day activity for some of them with an anti-poverty community organization, helped them uncover their prejudices and assumptions about poverty.

## Methods

### Study design

This study was part of a larger research program aimed at identifying family physicians’ competencies in providing care to socioeconomically disadvantaged patients in Quebec, Canada. Photovoice was first developed by Wang and Burris [22] in the 1990s among women in China’s Yunnan Province. The methodology has been applied to various populations since then, including women, children, immigrants, and the elderly, in different parts of the world, including the United States and Canada. There are several examples of projects that use photography to enhance dialogue and social change in the healthcare field [23]. According to Wang and Burris [24] reflexive discussion of photographs facilitates a critical dialogue about specific issues of concern. Photovoice is a participatory action research approach because it is aimed at generating concrete changes that may involve policy-makers and larger sectors of the population [22].

### Recruitment and participants

The first author of the present article is a sociologist and qualitative health researcher who supervises medical residents in their scientific research training. Medical supervisors in the university’s academic primary care unit invited the researcher to present her research projects and interests to medical residents. She briefly presented her research program to the cohort of medical residents (approximately 25 residents) and invited them to explore, under her supervision, a specific research question regarding family medicine practice and poverty.

Four family medicine residents expressed interest in participating in a research project with her and her postdoctoral fellow. The two researchers proposed to collaborate with the medical residents and their clinician supervisors on a project using photovoice. The four medical residents and two medical supervisors (one physician and one psychologist) were then recruited and agreed to participate on a voluntary basis. The medical residents were relatively similar to their counterparts in terms of ethnic and socio-economic background: they were born in Quebec and were from middle- to high-income families. Among the four, one resident was older than the others and had past professional life experience in an area other than medicine. Two social science researchers—a sociologist and an anthropologist —were also part of the photovoice group, for a total of eight participants.

### Design and procedure

We conducted this study in an academic primary care unit of the Faculty of Medicine at Sherbrooke University, Quebec, Canada. The university’s research ethics committee approved the project, and participants signed a consent form. Our study, conducted in 2010–2011, consisted of six three-hour meetings over seven months. All meetings were recorded and transcribed for analysis. After each meeting, we took field notes and researchers discussed their interpretations to deepen the understanding of the experiential learning.

In the first meeting, the researchers provided training on the photovoice methodology, related concepts, and goals. In line with the research objectives, the medical residents formulated a research question: What are the barriers between medical residents and socioeconomically disadvantaged persons? The second author, a visual anthropologist, prepared the team to use photography as a means of responding to the research question and to begin a reflexive process. All eight participants were invited to take photos, over a four-week period, that responded in some way to the research question, to foster a reflexive approach in all the participants. Participants were free to take as many photos as they wanted to, but were asked to bear in mind that they would need to choose five photos to share with the group at the next meeting. Participants could take photos anywhere, as long as they preserved the security and the anonymity of any persons they photographed on the street. A majority of the photos were taken outside, in the city where the participants were living. The photographs were very diverse: an empty fridge, a pharmacy shelf showing drug price labels, a wrecked car, a homeless shelter, etc.

The second meeting took place four weeks later, to allow participants time to reflect on the research question and take photos. At this meeting, each participant presented five photos, explaining the reasons for taking them and for sharing them with the group. After participants had shared their interpretations of their own photos, the others were invited to comment.

In the four remaining meetings, the medical residents analyzed the data using qualitative research methodology. During this analysis phase, two of them also participated on a voluntary basis in a one-day activity with an anti-poverty community organization, ATD Fourth World, which involved selecting photos that reflect happiness. They listened as socioeconomically disadvantaged persons explained why they selected certain photos and what happiness was, from their perspective. Following this activity, the participants shared critical reflections with the other photovoice participants.

The researchers trained the residents in qualitative analysis. Assisted by the researchers, the residents themselves created a coding scheme and coded the transcripts of the second meeting (at which the photos were exhibited). The analysis progressed between meetings through emails and telephone calls among researchers and residents. With the researchers’ support, the residents analyzed data, did peer debriefing, created a table synthesizing the data, and discussed interpretations with researchers.

At the end, the residents gave an oral presentation of the results to medical residents, supervisors, and managers at the Annual Research Day of the Faculty of Medicine. They received two significant awards in recognition of their work.

### Data analysis

All meetings were audio-recorded and transcribed. Data analysis consisted of data coding, peer debriefing, and validation of interpretation. The medical residents coded and analyzed the second meeting, and a visual anthropologist coded and analyzed the transcripts of all the meetings in collaboration with two researchers. The researchers validated the coding and were involved in data interpretation through reading transcripts and independently coding the data and by attending regular meetings. An interpretive content analysis framework [24] was used to interpret the data. Our aim was to identify verbal exchanges that indicated reflexivity and critical consciousness directly sparked by the photovoice project. Data analysis involved three interrelated steps: 1) repeated reading of each discussion transcript by the medical residents and the researchers to “get a sense of the whole” [25]; 2) extraction from the transcripts of storylines discussed; and 3) establishing links between storylines, which became the core of the analysis section of this article. In this study, data reliability was maintained by data triangulation, participant validation, supervision, and peer review.

## Results

The medical residents acknowledged at the outset that they had, in their words, “few tools” to respond to the situations of socioeconomically disadvantaged patients. Their involvement in the photovoice project with supervisors and researchers who study poverty improved their knowledge and raised their awareness of issues of poverty and primary care. Residents felt encouraged to consider adopting a different care approach and trying to overcome barriers between themselves and their socioeconomically disadvantaged patients. In the photovoice project, medical residents underwent a three-phase learning process that could be summarized as: (1) engaging reflexively; (2) break(ing) through; and (3) taking action. The characteristics of each phase are described below.

### Phase1: engaging reflexively with poverty

The process of reflexivity started in the first meeting. The residents expressed discomfort with scrutinizing their experience through a photovoice project (thereby becoming what they called “photovoice subjects”). They learned that photovoice projects are often conducted among vulnerable populations, and did not see themselves in that category. However, after long and intense discussion with supervisors and researchers, they admitted their lack of knowledge of how to interact with socioeconomically disadvantaged persons. They reported feelings of incompetence and powerlessness that prevented them from addressing certain issues with socioeconomically disadvantaged patients. They realized that, in that sense, they were somewhat in a position of professional vulnerability. This moment of collective awareness prompted them to commit to the photovoice process. The project also encouraged them to explore what poverty meant to each of them and how they could represent it visually.

Defining poverty from the participants’ perspectives was a key part of the process throughout, but reached a turning point in the second meeting when one participant presented her final photograph, that of a 50-year old man who had given up his medical practice:

This is a nice picture because this gentleman … decided to quit [his job] to live in a state of voluntary simplicity. … He was tired of being super rich and having 56,000 things to do. So, I just wanted to introduce the notion that in a very small percentage of cases, it’s possible that some people choose to live in poverty. … Poverty is just that: it is the absence of choice, the absence of means, that puts you in a particular situation. … But when you have the option to be able to get out of it, then it’s different.

The discussion around this picture and the notion of “real poverty” had a major impact on the resident’s level of sensitivity concerning what it is to experience poverty. The process of defining poverty also touched upon their own feelings and personal stories. One striking case emerged when a medical resident revealed, through her photos, that one of her close relatives with children was living in poverty. In fact, the perception of what poverty means became both a personal and a professional issue for all participants. Residents spoke of the tension between “doing something” and “being powerless” when confronted with certain aspects of poverty. They expressed their desire to provide good-quality care to socioeconomically disadvantaged patients but said they lacked tools and resources to respond to needs related to patients’ precarious economic situation.

The photovoice project allowed residents to reflect on their own prejudices and fears concerning poverty. Admitting publicly that one has prejudices is not easy, but the openness of participants who shared similar thoughts provided a safe environment for such confidences. Several forms of prejudices were uncovered during the discussions. They included the ideas that “poor people should not have leisure activities that cost money because they can’t afford it”, that socioeconomically disadvantaged patients abuse the healthcare system, that they look down on rich people and are envious of them, and that poor and rich people live in two different worlds.

### Phase 2: break(ing)through

Zigon [26] argues that moral breakdowns are significant ethical moments to consider when looking at issues of morality. As a methodological approach encouraging reflexivity, photovoice invites people to step back and take positions on certain issues and problems. As such, it has the potential to provoke moral breakdowns, or moments of reflexive questioning. Break(ing)through, or making breakthroughs, refers to moments of self-critical awareness during which one acknowledges one’s biases, prejudices, and social position in relation to others. It is destabilizing, in that it fosters introspection based on ethical principles. An ethical imperative to solve the breakdown—break(ing)through— is placed on the person or group experiencing this moment, after which, according to Zigon [26], they are able to return to an “unreflective everydayness”, a much less troublesome state of being.

It was during residents’ discussions of their photos that most of the breakthroughs generated by moral breakdowns occurred. These breakthroughs were facilitated by interactive feedback from the professionals involved in the project, which fostered two interrelated results: 1) the residents accepted that they could be photovoice subjects; and 2) they acknowledged having emotions that could be discussed openly by naming the barriers and prejudices that hindered their effectiveness with socioeconomically disadvantaged patients.

The first breakthrough occurred during the first meeting. Residents argued vehemently that they could not be the subjects of a study:

… It’s rare that we, doctors, are included in that category [of vulnerable people], which makes us uncomfortable with this topic. However, it is somewhat embarrassing to say that we are uncomfortable, and have grey zones and areas where we feel powerless. So … I think we become a photovoice subject, if we’re forced to face our own prejudices.

The professional team’s mentoring of the residents was a crucial part of the photovoice project. Constant probing from the supervisors brought the residents to accept that they could become the subjects of their own questioning and of a qualitative project that would not necessarily give them yes/no answers. With guidance, they were afforded time and space in which to identify and name, through photos and guided conversations, the barriers, obstacles, prejudices, and stereotypes they each had related to poverty and socioeconomically disadvantaged persons.

The changes that occurred during the process were enabled by open-ended discussions that encouraged the residents to express themselves—an experience that runs counter to their training, which teaches them to make decisions based on objective facts and rational observations. Despite initial resistance, many “breakthroughs” occurred in the residents’ conversations, mainly facilitated by the supervisors’ mentoring. They encouraged the residents to express themselves openly and to pursue their reasoning further. For instance, at the end of the project the residents all acknowledged the social distance that prevails between them and persons who live in poverty. They realized how hard it could be for persons living in poverty to deal with the biomedical language of doctors if they are not familiar with it. Also, residents recognized they may be predisposed to making negative initial judgments concerning people in poverty:

There is always a small voice of prejudice in the back of my head saying that a person in a situation of poverty […], I have the feeling that these are people who tend to use drugs, drink a lot of alcohol, and smoke.

### Phase 3: taking action

Being reflexive in relation to a situation was empowering for all the participants, but particularly for the medical residents. One resident reported that discussing issues of poverty with the other participants empowered her to ask one of her patients personal questions she would not have to ask before, which opened up the possibility of exploring what resources in the community were available for her patient. She directly attributed her new professional behavior to the reflexive process fostered by the photovoice project. Instead of ignoring the problem, she addressed it directly, without shouldering all the responsibility. Residents expressed feeling better equipped to serve patients with health problems caused in part by poverty. Also, after medical residents presented their data to the Faculty members, a “social competence” component was incorporated into the spectrum of competencies that medical residents should acquire during their training and a course on poverty was developed and given to medical residents.

## Discussion

This study explored residents’ perceptions and experience in relation to providing care for socioeconomically disadvantaged persons. It also examined how residents’ involvement in a photovoice project helped them uncover their prejudices and assumptions concerning poverty. In this project, residents underwent a three-phase learning process of: (1) engaging reflexively; (2) break(ing)through; and (3) taking action. Sharing experiences involved lengthy conversations in which participants responded to photos presented by others, made connections with other participants’ comments, and highlighted differences and similarities. In fact, most “individual” photos became “group” photos as they were appropriated by each of the participants, who were stimulated by the photos to reflect on their own concerns.

As in other photovoice projects, this research generated a reflexive process that led to sustained critical consciousness and constituted “transformative learning” [27,28]. As noted by Sandars [29], the medical residents reported undergoing transformations in their perceptions of themselves and of the world triggered by a process of introspection during which feelings of powerlessness, sadness, shame, and anger emerged.

We believe the medical residents’ experience with the photovoice project encouraged them to take poverty into serious consideration in their practice. The entire photovoice project, encompassing three phases, was a learning process for all participants. Residents’ concrete actions in direct response to the project could be seen as evidence that it was an effective teaching tool. As reported by Carlson, Engebretson, and Chamberlain [17], photovoice can be a means for expanding social consciousness and triggering social change, because its use fosters both reflection and action.

We acknowledge that the research was limited by the small number of participants. Small sample size in photovoice research initiatives is not uncommon because of the time requirements and the nature of the participants’ involvement. For example, in their oft-cited article on African-American mothers, Killion & Wang [30] had five participants. Another limitation of our study relates to the fact that the medical supervisors fulfilled multiple roles with the medical residents. A posteriori we considered it to be an asset for our data collection, because the supervisors contributed valuable input to the photovoice meetings. However, we observed that power relations could arise among the participants because the supervisors were involved in the residents’ evaluations.

By involving both medical residents and supervisors in this photovoice study, we made one of the first attempts in a medical academic milieu to involve healthcare professionals in identifying ways to improve physicians’ competencies in delivering care to socioeconomically disadvantaged patients. The study and its outcomes also generated momentum for changes to the family medicine curriculum at a teaching university. Thus, to improve the knowledge and skills of future primary care doctors and incorporate a “social competence” component into residency programs, we recommend developing an experiential learning approach by giving residents opportunities to be involved in underserved community activities and in reflexive discussion with medical supervisors.

In conclusion, photovoice appears to be a promising and innovative teaching approach in medical education, especially for medical residents. More specifically, our participatory research project helped future physicians become aware of their prejudices and motivated them to acquire skills for delivering healthcare to socioeconomically disadvantaged patients. Photovoice can be an effective tool for raising health professionals’ awareness of socioeconomic realities of their patients.

## Conclusions

This study highlights the importance of providing medical training on issues related to poverty and increasing awareness about social inequalities in medical education to counteract prejudices toward socioeconomically disadvantaged patients. Future studies should examine which elective courses and training could provide suitable tools to clinicians to improve their competence in delivering care to socioeconomically disadvantaged patients.
